# Relaxant Effect of *Urginea maritima* on Tracheal Smooth Muscle Mediated by the Effect on Beta-2 Adrenergic, Muscarinic Receptors and Calcium and Potassium Channels

**DOI:** 10.1155/2021/6637990

**Published:** 2021-04-20

**Authors:** Hamideh Kazemi Rad, Arghavan Memarzia, Fatemeh Amin, Mohammad Hossein Boskabady

**Affiliations:** ^1^Applied Biomedical Research Center, Mashhad University of Medical Sciences, Mashhad, Iran; ^2^Department of Physiology, Faculty of Medicine, Mashhad University of Medical Sciences, Mashhad, Iran; ^3^Non-Communicable Diseases Research Center, Rafsanjan University of Medical Sciences, Rafsanjan, Iran; ^4^Department of Physiology and Pharmacology, School of Medicine, Rafsanjan University of Medical Sciences, Rafsanjan, Iran

## Abstract

*Urginea maritima* (*U. maritima*) showed anti-inflammatory, antioxidant, antibacterial, diuretic, vasodilatation, and wound-healing effects on fungal infections, cardiac disorders, digestive disorders, rheumatoid disease, and respiratory disorders such as bronchitis, bronchial nosocomial infections, and severe cough. To examine the bronchodilatory effect of *U. maritima*, the relaxant effect of its extract on rat tracheal smooth muscle (TSM) and its possible mechanism was examined in this study. Male Wistar rats' TSM were divided into eight groups (*n* = 8 in each group). Four of these groups were TSM tissues, contracted with KCl (60 mM) incubated with atropine, glibenclamide, and indomethacin and nonincubated TSM, while the other four groups were TSM tissues contracted with methacholine (10 *μ*M) for 5 min, incubated with propranolol, chlorpheniramine, and diltiazem and nonincubated TSM. Cumulative concentrations of *U. maritima* extract (12.5, 25, 50, 100, 20, and 400 *μ*g/ml) were then added to organ bath every 5 min. Theophylline (0.2, 0.4, 0.6, and 0.8 mM) as positive control and saline (1 ml) as negative control were also examined in nonincubated tissues. A concentration-dependent relaxant effect of *U. maritima* on nonincubated TSM contracted with KCl (60 mM) or methacholine (10 *μ*M) (*p* < 0.01 and *p* < 0.001) was observed. The relaxant effects of *U. maritima* extract in the incubated tissues with glibenclamide, propranolol, diltiazem, atropine, and chlorpheniramine were significantly lower than those in the nonincubated tissues (*p* < 0.05 to *p* < 0.001). EC_50_ values of *U. maritima* extract in the incubated TSM with glibenclamide, propranolol, diltiazem, and atropine were significantly higher than those in the nonincubated tissues (*p* < 0.05 for diltiazem-incubated tissues and *p* < 0.001 for other cases). *U. maritima* extract displayed considerable relaxant effect on TSM comparable to the effect of theophylline. Beta-2 adrenoceptor stimulation and muscarinic receptor inhibition as well as potassium opening and calcium channels blocking effects are the possible mechanisms for the relaxant effects of the plant.

## 1. Introduction

One of the most important chronic inflammatory diseases in the world is asthma with considerable morbidity. Asthma is characterized by pathological changes in the lung, like increased mucosa secretion, airway hyperresponsiveness, infiltration of inflammatory cells, and smooth muscle hyperplasia [1]. Over the past 30 years, there has been an increase in the number of patients with asthma, and 250,000 people die from this disease each year. The treatment of this disease is very costly and the direct and indirect costs of the asthma are globally on the rise [2]. The precise mechanism of asthma pathophysiology and the role of biochemical intermediates involved in asthma are not yet known, but, based on available information, leukotrienes, prostaglandins, histamine, nitric oxide, and type II immune response cytokines are among the most important mechanisms involved in the pathophysiology of asthma. These mechanisms cause airway inflammation as well as bronchospasm in asthmatic patients [3]. The adrenoceptor and cholinergic pathways affected the airway smooth muscle tone. Cholinergic control is performed by the vagal reflex and through stimulatory receptors located below the mucous membranes of the large respiratory tract and upper respiratory tract. The stimulation of these receptors by inhaled stimulants or inflammation causes obstruction of large airways. Androgen intermediates like histamine and prostaglandins may cause bronchial smooth muscle contraction directly or reflexively through stimulation of receptors [4].

The tendency to use treatments with minimizing side effects such as the use of medicinal herbs which may cause a decrease in drug resistance has increased in recent years [5]. *Urginea maritime* (*U. maritima*) is a flowering plant of the Asparagaceae family and the Scilloideae subfamily, known as squill, sea squill, and onion, growing in the Mediterranean, North Africa, and India [6, 7]. *U. maritima* contains a large number of glycosides, type bufadienolide, where scillaren A is an important component of all glycosides. *U. maritima* also contains an aclicon called scillaridin A and small amounts of other cardiac glycosides, such as glucoscillaren A, proscillaridin A [8], other flavonoids, fatty acids, anthocyanins, and related carbohydrates [9, 10]. *U. maritima* plant has been widely used in cardiac disorders and fungal infections and as a diuretic agent, and the fresh *U. maritima* extract is more active than the dried fruits [11]. Constituents of methanolic extract of *U. maritima* exhibited antioxidant and antibacterial activity [12]. A previous document exhibited biological activity of *Urginea* species [13]. The plant also displayed antibacterial activity, inhibition of the growth of an ascites tumor [14], and cardiotonic and diuretic effects [15]. The potent digitalis-like cardiac effect of the plant was described for centuries [16], and it showed heart stimulatory and diuretic effects [17]. Fresh bulbs of this species are used to accelerate wound-healing, as well as in digestive disorders and rheumatoid disease [18]. The effect of this plant on the treatment of cancer was also shown [19]. It showed cytotoxicity against human breast carcinoma cells (MCF-7) in vitro [20]. *U*. *maritima* extract also caused peripheral vasodilatation in anesthetized rabbits [21] and it showed similar effect to that of digitalis on the heart. *U*. *maritima* is traditionally used to treat bronchitis, bronchial nosocomial infections, severe cough, and edema [22, 23].

Therefore, to examine the bronchodilatory effect of *U. maritima*, this study sought to investigate the relaxant effect mechanisms of action of its extract on tracheal smooth muscle (TSM) in Wistar rats.

## 2. Materials and Methods

### 2.1. Preparation of the Extract

*U. maritima* was purchased from a market in Mashhad, Iran, in October 2018 and identified by Dr. Rakhshandeh, Pharmacological Research Center of Medicinal Plants and Department of Pharmacology, Faculty of Medicine, Mashhad University of Medical Sciences, Mashhad, Iran. *U. maritima* extract was prepared by peeling, weighed (50 g), and soaked in 70% ethanol (ethanol 96°, Taghtir Khorasan Co., Iran) at 40°C for 72 hours while shaking constantly. The extract was dried by rotary evaporator at 50°C to obtain a yield of 12% and the required concentrations were prepared.

### 2.2. Animals and Experimental Groups

Sixty-four male Wistar rats (weight, 200–250) were kept in a standard condition, 22 ± 2°C temperature, 12 h light/dark cycles, and free access to standard diet and tap water in the Animal House, School of Medicine, Mashhad University of Medical Sciences, Mashhad, Iran. The study was approved by the Ethics Committee of Mashhad University of Medical Sciences (#961800). All experiments on animals were done according to National Laws regarding the use and care of laboratory animals. Animals were divided into eight groups (*n* = 8 in each group) as shown in Table 1.

### 2.3. Tissue Preparation

The rats were sacrificed after anesthetizing by 1.6 g/kg intraperitoneal (i.p.) administration of urethane and their chests opened. Tracheal rings of rats containing three cartilages were prepared from the middle section of trachea as previously described [24] and mounted in a 10 ml organ bath containing Krebs-Henseleit solution supplied with 95% O_2_ and 5% CO_2_ and tissue responses were measured using an isometric transducer (MLT0202, AD Instruments, Australia) connected to a power lab system (Power Lab 8/30, ML870, AD Instruments, Australia) exactly as previously described [24–26].

### 2.4. Examination of Smooth Muscle Relaxant Effect of Plant Extract

TSM was contracted by KCl (60 mM) (Merck Chemical Ltd., Germany) or methacholine (10 *μ*Μ) (Sigma Chemical Ltd., UK) [26]. It was well established that KCl contracts tracheal smooth muscles by depolarizing the smooth muscle cells and methacholine contracts them by muscarinic receptor stimulation [24, 26]. After 5 minutes, cumulative concentrations of extract of *U. maritima* (12.5, 25, 50, 100, 20, and 400 *μ*g/ml) [27] and theophylline (0.2, 0.4, 0.6, and 0.8 mM) as positive control or saline (1 ml) as negative control were added to organ bath every 5 minutes [25, 26].

The reduction of contraction produced contractile agents (KCl or methacholine) due to the fact that each concentration of *U. maritima* extract and theophylline in proportion to maximum contractile response was calculated and considered as percent relaxation response [24]. The relaxation concentration response curves were prepared, and the extract concentration causing 50% of maximum relaxation effect (EC_50_) was measured from concentration response curve as previously defined [24–26, 28–31]. In incubated tissues with rightward shift in the concentration-response curve of the relaxant effect of the plant, the concentration ratio minus one (CR-1) was also estimated as (EC_50_ in the incubated tissues/EC_50_ in nonincubated tissues)-1.

In two groups, the relaxant effect of *U. maritima* extract was examined on KCl- or methacholine-contracted on nonincubated TSM to evaluate the possible relaxant effect of the plant and its possible effect on potassium channels and muscarinic receptors, respectively. In addition, in six groups, the relaxant effects of the extract on incubated TSM tissues with atropine, glibenclamide, and indomethacin, contracted by KCl, as well as incubated TSM with propranolol, chlorpheniramine, and diltiazem, contracted by methacholine, were examined to evaluate different possible mechanisms responsible for the relaxant effect of *U. maritima* extract as described in Table 1. The duration of the examination of the relaxant effect of the extract including mounting the tracheal ring in the organ bath, tissue equilibration, TSM contraction, and evaluation of the relaxant effect in each experiment was about 100 minutes.

### 2.5. Statistical Analysis

Statistical comparisons were performed using InStat software. The data was presented as mean ± standard error of the mean (SEM). Comparisons were performed using ANOVA followed by Tukey's multiple comparisons test and *p* < 0.05 was considered as a significant criterion.

## 3. Results

### 3.1. The Relaxant Effect of *U. maritima* Extract on TSM Contraction Induced by Methacholine in Nonincubated and Incubated Tissues

Concentration-dependent and significant relaxant effects of the extract of *U. maritima* and theophylline were seen on TSM contracted by methacholine (*p* < 0.05 for the second extract concentration and *p* < 0.001 for all theophylline and higher extract concentrations).

**Figure 1:**
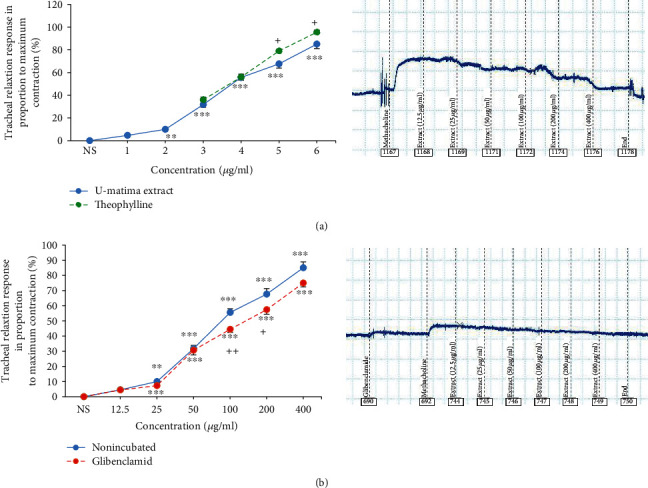
Concentration-response relaxant effects (mean ± SEM) of theophylline and *U. maritima* extract in nonincubated TSM contracted by 10 *μ*M methacholine (*n* = 7). 1, 2, 3, 4, 5, and 6 in *X*-axis display six concentrations of the extract (12.5, 25, 50, 100, 20, and 400 *μ*g/ml) and 3, 4, 5, and 6 display theophylline concentrations (0.2, 0.4, 0.6, and 0.8 mM) and (b) concentration-response relaxant effects (mean ± SEM) of theophylline and *U. maritima* extract in glibenclamide-incubated TSM (1 *μ*M, *n* = 8). ^∗∗^*p* < 0.01 and ^∗∗∗^*p* < 0.001 compared to the effect of saline (NS), ^+^*p*<0.05 in panel (a) indicates comparison between the effect of theophylline and that of the extract. ^+^*p*<0.05 and ^++^*p*<0.01 in panel (b) show the comparison of the effect of the extract between incubated and nonincubated tissues. ANOVA with the Tukey–Kramer post hoc test was used for statistical comparison.

The relaxant eﬀects of two higher concentrations of *U. maritima* extract (200 and 400 *μ*g/ml) were significantly less than the relaxant eﬀects of the two higher concentrations of theophylline (*p* < 0.05 for both cases) (Figure 1(a)).

**Figure 2:**
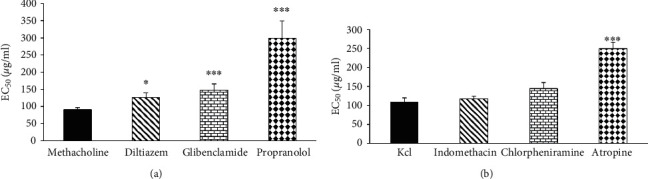
EC_50_ values of *U. maritima* extract-induced TSM relaxation in nonincubated and incubated TSM with various agents and contracted with methacholine (a) or KCl (b). ^*∗*^*p*<0.05 and ^∗∗∗^*p* < 0.001 compared to nonincubated tissues. ANOVA with the Tukey–Kramer post hoc test was used for statistical comparison.

Different concentrations of *U. maritima* extract showed significant relaxant effects on TSM in incubated tissue with glibenclamide (*p* < 0.001 for 5 last concentrations). However, in incubated tissues with glibenclamide, the relaxant effects of 100 and 200 *μ*g/ml concentrations of the extract were significantly lower than those in the nonincubated TSM (*p* < 0.001 and 0.05 for 100 and 200 *μ*g/ml concentrations, respectively) (Figure 1(b)). EC_50_ values of the *U. maritima* extract for its relaxant effect in incubated TSM with glibenclamide were signiﬁcantly higher than those in nonincubated tissues (Figure 2(a)).

A rightward shift in the concentration-response relaxation curve of the *U. maritima* extract was observed; in glibenclamide-incubated TSM compared to nonincubated tissues, a maximum response was achieved. The (CR-1) value of the extract in incubated TSM with glibenclamide was 0.6 ± 0.2.

**Figure 3:**
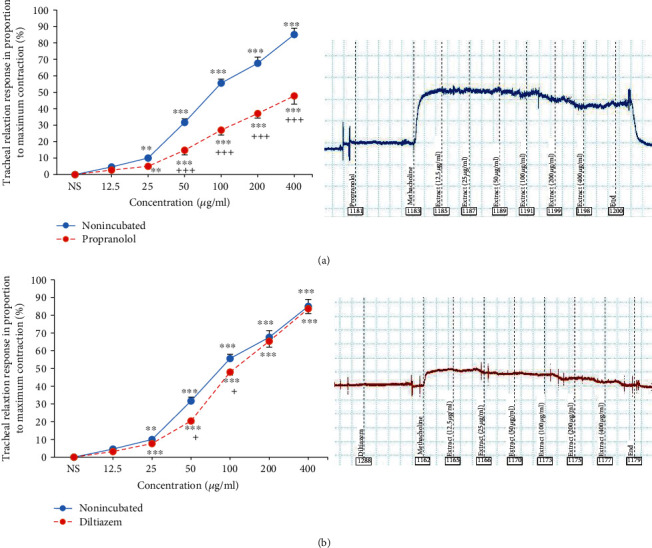
Concentration-response relaxant effects (mean ± SEM) of the *U. maritima* extract on 10 *μ*M methacholine-induced contraction of TSM in nonincubated and propranolol-incubated (1 *μ*M) (a) and diltiazem-incubated TSM (1 *μ*M) (b) (*n* = 8 for all groups). ^∗∗∗^*p* < 0.001, compared to the effect of saline (NS), ^+^*p*<0.05, compared to the effect of the extract on nonincubated tissues. ANOVA with the Tukey–Kramer post hoc test was used for statistical comparison.

The relaxant eﬀects of different concentrations of *U. maritima* extract on incubated tissue with propranolol were significantly higher than the effect of saline (*p* < 0.01 for the second and *p* < 0.001 for higher extract concentrations). The relaxant effects of four higher concentrations of the extract in incubated TSM with propranolol were significantly lower than those in the nonincubated tissues (*p* < 0.001 for all cases, (Figure 3(a)). EC_50_ value of the *U. maritima* extract for its relaxant effect in incubated tissues with propranolol was signiﬁcantly higher than that in the nonincubated TSM (*p* < 0.001) (Figure 2(a)). A rightward shift in concentration-response relaxation curve of the *U. maritima* extract was observed in propranolol-incubated TSM compared to nonincubated tissues but the maximum response was not achieved. The (CR-1) value of the extract in incubated TSM with propranolol was 2.3 ± 0.4.

Different concentrations of *U. maritima* extract caused significant relaxant eﬀects in incubated tissues with diltiazem compared to the effect of saline (*p* < 0.001 for 5 last concentrations). The relaxant eﬀects of 50 and 100 *μ*g/ml of the extract in incubated tissue with diltiazem were significantly lower than those in the nonincubated TSM (*p* < 0.05 for both cases) (Figure 3(b)).

EC_50_ values of the *U. maritima* extract for its relaxant effect in incubated TSM with diltiazem were signiﬁcantly higher than those in the nonincubated tissues (*p* < 0.05) (Figure 2(a)). A rightward shift in concentration-response relaxation curve of the extract was observed in diltiazem-incubated TSM compared to nonincubated tissues and the maximum response was achieved. The (CR-1) value of the extract in incubated TSM with diltiazem was 0.43 ± 0.1.

### 3.2. The Relaxant Effect of *U. maritima* Extract on TSM Contraction Induced by KCl in Nonincubated and Incubated Tissues

**Figure 4:**
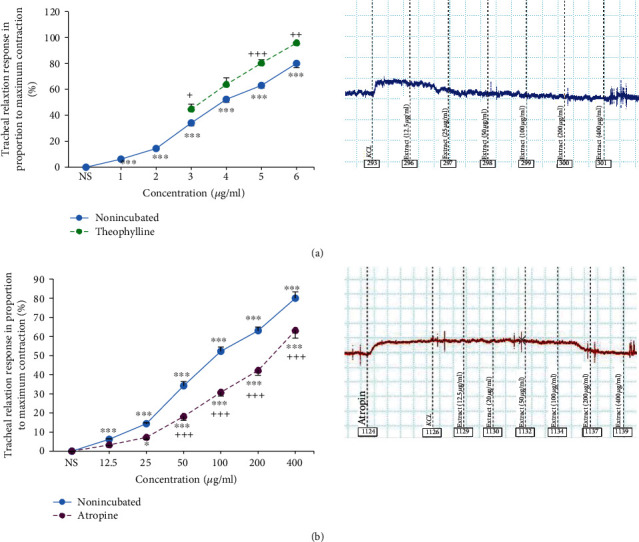
Concentration-response relaxant effect (mean ± SEM) of (a) theophylline and *U. maritima* extract in nonincubated TSM contracted by 60 mM KCl (*n* = 8). 1, 2, 3, 4, 5, and 6 in *X*-axis show six concentrations of the extract (12.5, 25, 50, 100, 20, and 400 *μ*g/ml) and 3, 4, 5, and 6 show theophylline concentrations (0.2, 0.4, 0.6, and 0.8 mM) and (b) atropine-incubated TSM (1 *μ*M, *n* = 6). ^*∗*^*p*<0.05 and ^∗∗∗^*p* < 0.001, compared to the effect of saline (NS), +*p*<0.05, ^++^*p*<0.01, and ^+++^*p*<0.001 in panel (a) indicate comparison between the effect of theophylline and that of the extract. ^+++^*p*<0.001 in panel (b) shows the comparison of the effect of the extract between incubated and nonincubated tissues. ANOVA with the Tukey–Kramer post hoc test was used for statistical comparison.

In nonincubated TSM contracted by KCl, the relaxant effects of all concentrations of *U. maritima* extract and theophylline were higher than the effect of saline (*p* < 0.001 for all cases except the low extract concentration). The relaxant effects of 0.2, 0.6, and 0.8 mM theophylline were significantly higher than those of the corresponding concentrations of the extract (*p* < 0.05 to *p* < 0.001) (Figure 4(a)).

The extract of *U. maritima* showed significant and concentration-dependent relaxant effects on incubated TSM with atropine (*p* < 0.05 for 25 *μ*g/ml and *p* < 0.001 for higher extract concentrations). The relaxant effects of four higher concentrations of the extract in incubated tissue with atropine were significantly lower compared to those in the nonincubated TSM (*p* < 0.001 for all cases) (Figure 4(b)).

EC_50_ values of the extract for its relaxant effect in incubated TSM with atropine were signiﬁcantly higher compared to those in the nonincubated tissues with atropine (*p* < 0.001) (Figure 2(b)).

A rightward shift in concentration-response curve of the extract was seen in atropine-incubated TSM compared to nonincubated tissues and the maximum response was achieved. The (CR-1) value of the extract in incubated TSM with atropine was 1.6 ± 0.2.

**Figure 5:**
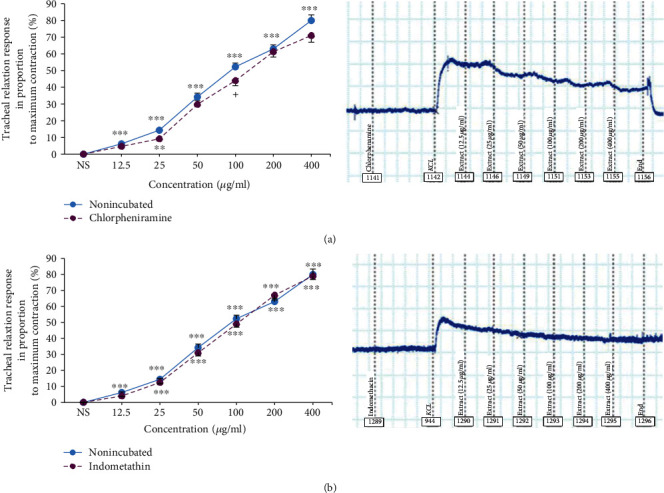
Concentration-response relaxant effects (mean ± SEM) of *U. maritima* extract on 60 mM KCl-induced contraction of TSM in nonincubated and chlorpheniramine-incubated (1 *μ*M) (a) and indomethacin-incubated TSM (1 *μ*M) (b) (*n* = 8 for all groups). ^∗∗^*p* < 0.01 and ^∗∗∗^*p* < 0.001, compared to the effect of saline (NS), +*p*<0.05 compared to the effect of the extract on nonincubated tissues. ANOVA with the Tukey–Kramer post hoc test was used for statistical comparison.

The relaxant effects in 5 higher concentrations of extract in incubated TSM with chlorpheniramine and indomethacin were significantly higher compared to the effect of saline (*p* < 0.05 for 25 *μ*g/ml in chlorpheniramine-incubated tissues and *p* < 0.001 for higher extract concentrations). There was no significant difference between the effects of different concentrations of extract in incubated TSM with chlorpheniramine and indomethacin and nonincubated tissues (Figures 5(a) and 5(b)).

### 3.3. Comparison of the Relaxant Effect of *U. maritima* Extract between TSM Contracted by Methacholine and KCl

**Figure 6:**
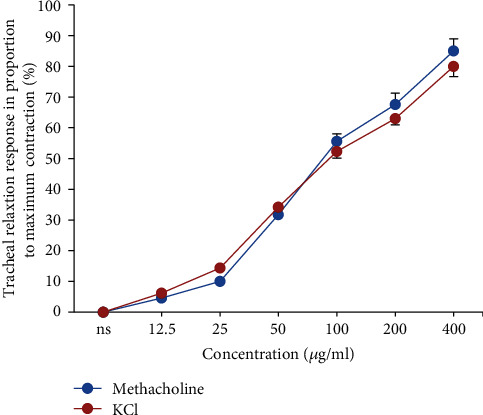
Concentration-response relaxant effects of the *U. maritima* extract in nonincubated tissues, contracted with 10 *μ*M methacholine (*n* = 8) and 60 mM KCl (*n* = 8). There was no significant difference between the relaxant effects of the plant on methacholine or KCl-induced TSM contraction. ANOVA with the Tukey–Kramer post hoc test was used for statistical comparison.

There was no significant difference between the relaxant effects of different concentrations of *U. maritima* extract between the TSM contracted by methacholine or KCl (Figure 6).

### 3.4. Correlations between Concentrations of the Extract of *U. maritima* and Theophylline with Their Relaxant Effects

The relaxant effects of theophylline and the extract were significantly correlated with their concentrations in all experimental groups (*p* < 0.001 for all cases) (Table 2).

## 4. Discussion

This study showed concentration-dependent relaxant effect of *U. maritima* extract in nonincubated TSM contracted by methacholine and KCl comparable to the effect of theophylline. The relaxant effect of *U. maritima* extract in nonincubated TSM contracted by methacholine and KCl was not significantly different. These results indicate a potent relaxant effect of the plant on TSM, which indicates its bronchodilatory effect in patients with obstructive pulmonary diseases. In fact, the effect of *U. maritima* in the treatment of respiratory diseases was indicated previously [23].

To examine the effect of *U. maritima* on *β*2-receptor [29], muscarinic [32], histamine (H1) [33] receptors, calcium channels [30], and potassium channels [34], ATP-sensitive potassium channels [34] and arachidonic acid metabolism [35] and their contribution in the relaxant effect of the plant were examined on tracheal smooth muscle incubated with propranolol, atropine, chlorpheniramine, diltiazem, glibenclamide, and indomethacin, respectively.

The relaxant effects of the extract in incubated tissues with propranolol, atropine, diltiazem, and glibenclamide were significantly lower than those in the nonincubated TSM. These results indicated the stimulatory effect of the plant on *β*2-adrenoceptor, inhibitory effect on muscarinic receptors, calcium channel blocking, and potassium channel opening effects, respectively. The EC_50_ values of the extract inducing relaxant effect in the incubated tissues with propranolol, atropine, diltiazem, and glibenclamide were also significantly higher compared to those in the nonincubated TSM. The higher EC_50_ values of the extract inducing relaxant effect in the incubated tissue also support the *β*2-adrenoceptor stimulation, muscarinic receptors inhibition, calcium channel blocking, and potassium channel opening properties of the plant. However, the maximum relaxant response was not obtained in incubated tissues with propranolol, which may indicate nonselective effect of the plant on *β*2-adrenoceptor [28]. The reason for the absence of maximum relaxant effect of the plant in incubated tissues with propranolol could be due to its effect on muscarinic receptors as well as calcium and potassium channels. The effect of another species of *U. maritima* extract on muscarinic receptors was demonstrated previously, which may support the effect of *U. maritima* on muscarinic receptor of TSM [24, 32]. Also, Memarzia and colleagues showed that the most important mechanism involved in relaxant effects of *Allium cepa* (*A. cepa*) extract was *β*2-adrenergic stimulatory and/or calcium channel [24], which may support the results of this study. A former study showed the relaxant activity of the extract of *Urginea indica* (another plant from Asparagaceae family) with the possible anticholinergic and Ca^2+^ antagonist mechanisms [27], which also supports the findings of the present study. However, the relaxant effects of *U. maritima* extract and its EC_50_ values in incubated tissues with chlorpheniramine and indomethacin were not significantly different from those in the nonincubated tissues. These results indicated the absence of the effect of the plant on histamine (H1) receptor [33] and arachidonic acid metabolism [35] pathways, ATP-sensitive potassium channels [34], and the contribution of these mechanisms to the relaxant effect of *U. maritima* extract on TSM.

This result showed relatively potent relaxant effect of *U. maritima* extract on TSM and the possible mechanisms of this effect for the first time. The mechanisms responsible for the relaxant effect of *U. maritima* extract on TSM are *β*2-adrenergic receptor stimulator, muscarinic receptors inhibition, calcium channel blocking, and potassium channels pathway opening effects or combinations of these mechanisms. The significant relaxant effect of *U. maritima* extract on TSM may indicate a bronchodilator effect for the *U. maritima* extract on obstructive pulmonary diseases.

## 5. Conclusions

In conclusion, this study displayed the potent relaxant effect of *U. maritima* on TSM comparable to the effect of theophylline, indicating its possible bronchodilatory property. Based on the results of this study, the possible mechanisms responsible for the relaxant effect of the plant on TSM are *β*2-adrenoceptor stimulation, muscarinic receptors inhibition, potassium channel opening, and calcium channel blocking properties.

## Figures and Tables

**Table 1:** Experiment groups. Trachea of incubated groups subjected to different channel blocker or antagonists in organ bath first followed by contraction of TSM by KCl or methacholine after 10 min.

Contractile agent	Condition	Incubating agent	Mechanisms	*n*
*KCl* (60 mM)	Nonincubated tissues	—	—	*n* = 8
	Incubated tissues	Atropine (1 *μ*M)	Muscarinic receptor inhibition	*n* = 8
		Indomethacin (1 *μ*M)	Cyclooxygenase inhibition	*n* = 8
		Chlorphenamine (1 *μ*M)	Histamine (H1) receptor inhibition	*n* = 8

*Methacholine* (10 *μ*M)	Nonincubated tissues	—	—	*n* = 8
	Incubated tissues	Diltiazem (5 *μ*M)	Calcium channel blocking	*n* = 8
		Glibenclamide (1 *μ*M)	Potassium channel opening	*n* = 8
		Propranolol (1 *μ*M)	Β_2_-adrenoceptor stimulation	*n* = 8

**Table 2:** Correlations between different concentrations and the relaxant effects of *U. maritima* extract and theophylline.

Contractile agents	Studied agents	Conditions	*R*	*p* value
*KCl*	*U. maritime*	Nonincubated	0.967	*p* < 0.001
		Atropine-incubated	0.936	*p* < 0.001
		Indomethacin-incubated	0.976	*p* < 0.001
		Chlorpheniramine-incubated	0.965	*p* < 0.001
	Theophylline	Nonincubated	0.899	*p* < 0.001
		Nonincubated	0.960	*p* < 0.001
		Diltiazem-incubated	0.963	*p* < 0.001

*Methacholine*	—	Glibenclamide-incubated	0.961	*p* < 0.001
	*U. maritima*	Propranolol-incubated	0.946	*p* < 0.001
	Theophylline	Nonincubated	0.966	*p* < 0.001

Data were presented as mean ± SEM.

## Data Availability

No data were used to support this study.
